# Effects of Exogenous *N*-Acyl-Homoserine Lactone as Signal Molecule on *Nitrosomonas Europaea* under ZnO Nanoparticle Stress

**DOI:** 10.3390/ijerph16163003

**Published:** 2019-08-20

**Authors:** Junkang Wu, Huan Gao, Jinyu Ye, Yan Chang, Ran Yu, Zhen Ding, Guangcan Zhu

**Affiliations:** 1 Department of Environmental Science and Engineering, School of Energy and Environment, Southeast University, Nanjing 210096, Jiangsu, China; 2 Key Laboratory of Environmental Medicine Engineering, Ministry of Education, Southeast University, Nanjing 210009, Jiangsu, China; 3 Jiangsu Province Center for Disease Control and Prevention, Nanjing 210009, Jiangsu, China

**Keywords:** ZnO nanoparticle, *Nitrosomonas europaea*, quorum sensing, stress tolerance, acyl-homoserine lactone

## Abstract

Despite the adverse effects of emerging ZnO nanoparticles (nano-ZnO) on wastewater biological nitrogen removal (BNR) systems being widely documented, strategies for mitigating nanoparticle (NP) toxicity impacts on nitrogen removal have not been adequately addressed. Herein, *N*-acyl-homoserine lactone (AHL)-based quorum sensing (QS) was investigated for its effects against nano-ZnO toxicity to a model nitrifier, *Nitrosomonas europaea*. The results indicated that AHL-attenuated nano-ZnO toxicity, which was inversely correlated with the increasing dosage of AHL from 0.01 to 1 µM. At 0.01 µM, AHL notably enhanced the tolerance of *N. europaea* cells to nano-ZnO stress, and the inhibited cell proliferation, membrane integrity, ammonia oxidation rate, ammonia monooxygenase activity and *amoA* gene expression significantly increased by 18.2 ± 2.1, 2.4 ± 0.9, 58.7 ± 7.1, 32.3 ± 1.7, and 7.3 ± 5.9%, respectively, after 6 h of incubation. However, increasing the AHL dosage compromised the QS-mediated effects and even aggravated the NPs’ toxicity effects. Moreover, AHLs, at all tested concentrations, significantly increased superoxide dismutase activity, indicating the potential of QS regulations to enhance cellular anti-oxidative stress capacities when facing NP invasion. These results provide novel insights into the development of QS regulation strategies to reduce the impact of nanotoxicity on BNR systems.

## 1. Introduction

In recent decades, engineered nanomaterials have become an emerging class of materials [1]. Diverse metallic nanoparticles (MNPs), displaying unique physical and chemical characteristics, have been broadly applied in commercial and industrial products, such as electronics, catalysts, pharmaceuticals, pigments, cosmetics, and textiles [2]. ZnO NPs (nano-ZnO), which have high water solubility, are among the most commonly used NPs, according to the Organization for Economic Cooperation and Development [3]. Due to the unique optoelectronic and catalytic properties [4], ZnO and nano-ZnO have been widely applied in many products, including solar cells, electronic sensors, antibacterial agents, photocatalysts, sunscreen, batteries, and glass [5,6,7]. The increasing use of MNPs and their inevitable emissions into the environment [8] have caused an increase in research focus on their bio-safety and risks to ecosystems [5,9].

As one of the most important environmental intermediates, the biological wastewater treatment plant is considered as a potential NP sink [10]. It has been estimated that the concentrations of nano-ZnO in the sewage-activated sludge are 31–200 mg/kg in Europe [11]. As a result, the retained nano-ZnO have been widely reported to decrease microbial community diversity, impair respiration rates of nitrifiers and heterotrophic bacteria, and inhibit nitrogen removal in biological nitrogen removal (BNR) systems [11,12]. In particular, ammonia-oxidizing bacteria (AOB) and ammonia oxidation activity have been shown to be vulnerable to nano-ZnO stress [11,13]. Given the foreseeable adverse effects of nano-ZnO on BNR processes, strategies for relieving NP toxicity to ensure stable BNR performance should be developed. Several studies have proposed physical or chemical methods to alleviate MNP toxicity, mainly by reducing the bioavailability of MNP and/or metal ion solubility [14,15] or adjusting environmental conditions (e.g., light intensity, substrate composition, and ion strength) [11,16]. However, strategies for enhancing microbial resistance to MNPs have seldom been reported.

Bacterial quorum sensing (QS) is a method of signalling transduction based on cell–cell communication processes. QS regulation mechanisms involve producing and sensing small signal molecules called autoinducers (AI), such as *N*-acyl-homoserine lactones (AHLs) [17]. When AHL concentrations reach a threshold value, bacterial communities coordinate their behaviour as a group by activating the expression of specific genes [18]. AHL-based QS regulation has been shown to commonly occur among nitrifiers in BNR systems [19]. For example, exogenous dosing of AHLs into BNR systems has been reported to favour the growth and metabolism of autotrophic nitrifiers [20], increase the ammonia oxidation rate (AOR) [21], and promote extracellular polymeric substance production [22]. In addition, QS regulation has been shown to actively participate in antibiotic resistance [17] and bacterial adaptive responses under graphene oxide stress [23]. Moreover, it has been found that Ag NPs could significantly decrease AHL concentrations and interrupt QS regulation in Gram-negative bacteria [24]. Therefore, we hypothesized that exogenous AHLs addition would be able to mediate NP biotoxicity.

The objectives of this study were to exam the impacts of a typical exogenous QS signalling molecule, *N*-3-oxo-hexanoyl-homoserine lactone (3-oxo-C_6_-HSL) addition on nano-ZnO toxicity to a model AOB, *Nitrosomonas europaea* [25], which produces at least three types of AHL, including C_6_-HSL, C_8_-HSL, and C_10_-HSL [26]. The effects of 3-oxo-C_6_-HSL on *N. europaea* cells’ proliferation rate, membrane integrity, AOR, and ammonia monooxygenase (AMO) activity were assessed when exposed to nano-ZnO in the short-term (6 h). Moreover, quantitative reverse transcription polymerase chain reaction (qRT-PCR) analysis was performed to quantify *amoA* gene expressions under nano-ZnO stress in cooperation with QS regulation.

## 2. Materials and Methods

### 2.1. Bacterial Strain

*N. europaea* ATCC 19718 strain was obtained from American Type Culture Collection and continuously cultured in a chemostat bioreactor at 25 °C in the dark. The working volume and hydraulic retention time in the chemostat were designed to be 3 L and 2.2 d, respectively. The pH in the reactor was controlled between 7.4–7.5 via automatic addition of 100 g/L sterile NaHCO_3_ with a Meller pH controller (Taiwan, China). The dissolved oxygen (DO) concentration was maintained around 2 mg/L by aeration of filter-sterilized air. The culture medium was the same as that used in our previous publication [27], which contained 20 mM NH_4_-N, 0.5 mM K_2_HPO_4_, 0.8 mM MgCl_2_, 10 mM 3-[4-(2-hydroxyethyl)-1-piperazine] propanesulfonic acid, 0.1 mM CaCl_2_, 2.4 μM EDTA–Fe^3+^, 1 μM CuCl_2_, 0.9 μM MnSO_4_, 0.4 μM Na_2_MoO_4_, 0.3 μM ZnCl_2_, and 0.02 μM CoSO_4_.

### 2.2. Chemicals and Nano-ZnO Characterization

C_6_-oxo-HSL and nano-ZnO were bought from Sigma-Aldrich (St. Louis, MO, USA). A C_6_-oxo-HSL stock solution (10 g/L) was prepared in dimethylsulfoxide (DMSO) [28], since AHLs are not soluble in water. The average primary and hydrodynamic diameter size (in DI water) of the NPs were determined using a JSM-6390 scanning electron microscope (SEM, Electronics Co., Ltd., Tokyo, Japan) and a Nano ZS90 analyser (Malvern, Worcestershire, UK), respectively as described previously [29].

### 2.3. Anti-Toxicity Experiment Design

We have previously reported that 10 mg/L (~0.1 mM) of nano-ZnO exerts significant toxicity effects on the metabolic activity of *N. europaea* [15,30]. Therefore, 0.1 mM of nano-ZnO was chosen to assess the anti-toxicity effects of exogenous QS signalling molecules.

A series of 100 mL cell culture samples were withdrawn from the chemostat reactor and incubated in 250 mL sterile flasks for batch testing. A sterile (NH_4_)_2_SO_4_ solution was added to all bottles to give a final NH_4_^+^-N concentration of 50 mg/L. One bottle was used as the control and it had no NPs or AHL added. Either nano-ZnO or C_6_-oxo-HSL was added to two of the bottles. To the other four bottles, both 0.1 mM nano-ZnO and 0.01, 0.1, 0.5, or 1 µM C_6_-oxo-HSL were added. Given the possible effects of DMSO on cell metabolic activity, only DMSO was added to another three bottles at 0.03, 0.17, or 0.33% (*v*/*v*, Table 1). After 6 h of incubation at 25 °C in the dark, the cultures were sampled for analysis. It can be seen from Table 1 that DMSO content up to 0.33% showed no significant (*p* > 0.05) adverse effects on cell density, membrane integrity, or AOR.

### 2.4. Analytical Methods

NH_4_^+^-N and NO_2_^−^-N concentrations were quantified using standard methods [31]. Bacteria were directly counted using a Z30000 Helber Counting Chamber (Hawksley, Lancing, UK) with a BX41 microscope (Olympus, Tokyo, Japan). Superoxide dismutase (SOD) activity, which is responsible for oxidative stress quenching, was measured using a SOD Assay Kit (Dojindo Molecular Technologies, Inc. Rockville, MD, USA), following the manufacturer’s instructions. Membrane integrity was determined using a LIVE/DEAD^®^ BacLight^TW^ Kit (Life Technologies, Waltham, MA, USA). Specific AMO enzyme activity, which is responsible for ammonia oxidation, was measured according to previously described procedures [30], and presented as NO_2_^−^-N production rate per unit of total protein. The concentrations of dissolved Zn^2+^ released from nano-ZnO were quantified using an AAnalyst 400 Atomic Absorption Spectrophotometer (PerkinElmer, Norwalk, CT, USA). Briefly, cell cultures were centrifuged at 9300 rpm to remove suspended cells and NPs. The supernatant was then filtered through a 0.22 µm membrane filter (Merck Millipore, Billerica, MA, USA) and acidized using 2% (*v*/*v*) HNO_3_ before Zn^2+^ measurement.

### 2.5. RNA Extraction and Quantitative Reverse Transcription Polymerase Chain Reaction (qRT-PCR)

Total RNA was extracted and purified using an RNeasy Mini Kit (Qiagen, Hilden, Germany) and then transcribed into cDNA using a QuantiTect^®^ Reverse Transcription Kit (Qiagen), according to the manufacturer’s instructions. The *amoA* gene, encoding AMO, and the 16S rRNA gene, were quantified using a CFX Connect Real-Time PCR Detection System (Bio-Rad Laboratories, Hercules, CA, USA). The specific primers used for the *amoA* and 16S rRNA genes and the amplification programs have been described previously [30] and are shown in Table 2. The expression level of *amoA* was normalized to that of the 16S rRNA gene.

### 2.6. Statistical Test

All experiments were performed in triplicate. The results are presented as mean ± standard deviation (*n* = 3). An unpaired two-sample *t*-test was used to assess statistical significance (*p* ≤ 0.05) between the test samples and the control.

## 3. Results

### 3.1. Nano-ZnO Characterization

**Figure 1:**
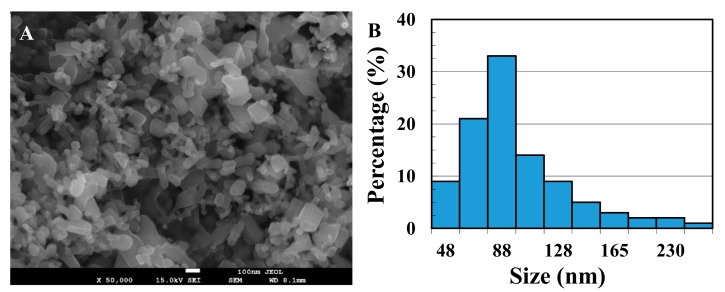
(**A**) Scanning electron microscope (SEM) imaging of nano-ZnO; (**B**) nano-ZnO size distribution.

The NPs’ average primary size was characterized to be 97 ± 38 nm by SEM imaging on the basis of measurements of 100 random particles (Figure 1). The hydrodynamic diameter size of 0.1 mM nano-ZnO in the water was 256 ± 55 nm.

### 3.2. Cell Growth and Membrane Integrity

**Figure 2:**
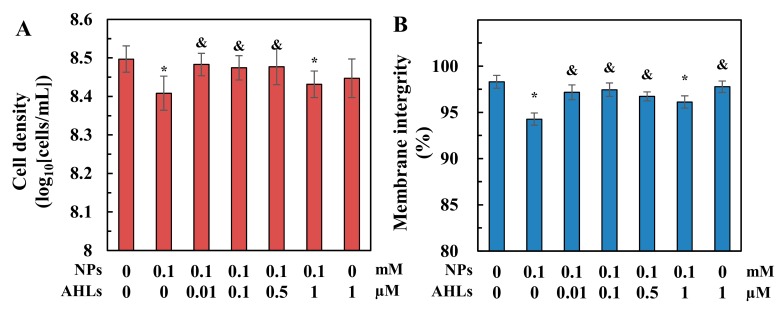
Effects of *N*-acyl-homoserine lactones (AHLs) dosing on *N. europaea* cells after 6 h of exposure to nano-ZnO: (**A**) cell density; (**B**) membrane integrity. ‘*’ and ‘&’ indicate significant differences (*p* ≤ 0.05) for test samples compared to normal cells and nano-ZnO-treated cells without AHL addition, respectively.

*N. europaea* cell density and membrane integrity remarkably decreased by 18.5 ± 1.8 and 4.4 ± 0.7%, respectively, after nano-ZnO stress (Figure 2). The addition of 0.01–0.5 µM C_6_-oxo-HSL significantly reversed the inhibition effects of nano-ZnO on cell growth and membrane integrity, which finally showed no significant differences (*p* > 0.05) compared to normal cells. However, C_6_-oxo-HSL doses as high as 1 µM had no effect on cell growth and membrane integrity impaired by nano-ZnO. Moreover, the addition of 1 µM C_6_-oxo-HSL into the *N. europaea* cultures slightly decreased cell concentrations and membrane integrity, but with no statistical differences (*p* > 0.05) compared to normal cells.

### 3.3. Ammonia Oxidation Rate (AOR), Ammonia Monooxygenase (AMO) Activity, and amoA Expression

**Figure 3:**
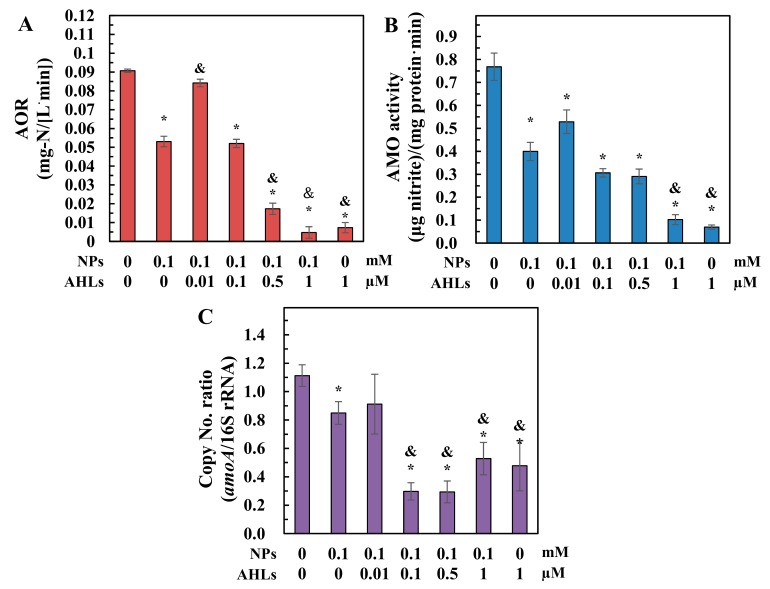
Ammonia oxidation activities in *N. europaea* cells after 6 h of exposure to nano-ZnO in the presence of different concentrations of *N*-acyl-homoserine lactones (AHLs): (**A**) ammonia oxidation rate (AOR); (**B**) ammonia monooxygenase (AMO) activity; (**C**) *amoA* expression. ‘*’ and ‘&’ indicate significant differences (*p* ≤ 0.05) for test samples compared to normal cells and nano-ZnO-treated cells without AHL addition, respectively.

The nano-ZnO induced inhibition of the ammonia oxidation activity in *N. europaea* cells was significantly attenuated after the addition of 0.01 µM C_6_-oxo-HSL. The cellular AOR, AMO activity, and *amoA* expression increased by 58.7 ± 7.1, 32.3 ± 1.7, and 7.3 ± 5.9%, respectively (Figure 3A–C), after 0.01 µM C_6_-oxo-HSL treatment. However, at concentrations of 0.1–1 µM, C_6_-oxo-HSL did not promote ammonia oxidation activity in *N. europaea* cells, but even inhibited AOR, AMO activity, and *amoA* expression with increasing concentrations of C_6_-oxo-HSL (Figure 3). Moreover, a single 1 µM dose of C_6_-oxo-HSL in normal *N. europaea* cultures significantly reduced AOR, AMO activity, and *amoA* expression (*p* ≤ 0.05).

### 3.4. Superoxide Dismutase (SOD) Activity

**Figure 4:**
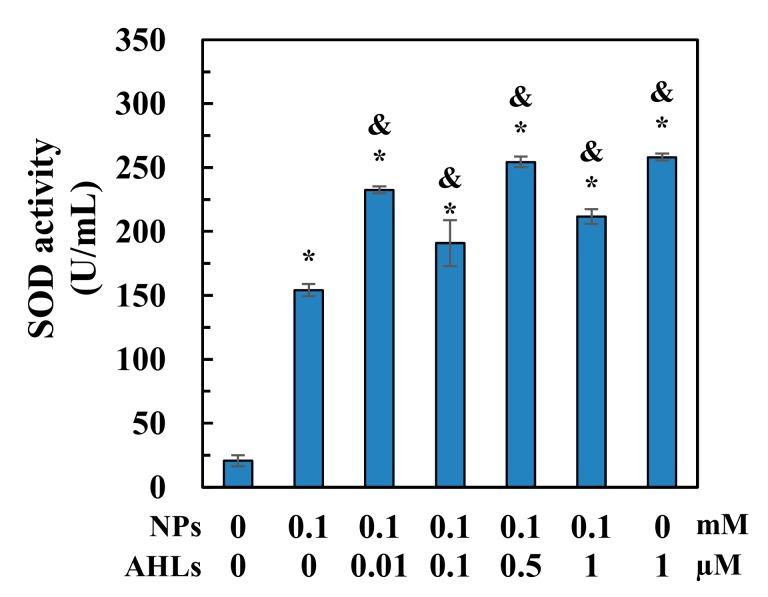
Effects of *N*-acyl-homoserine lactones (AHLs) dose on superoxide dismutase (SOD) activity in *N. europaea* cells after 6 h of exposure to nano-ZnO. ‘*’ and ‘&’ indicate significant differences (*p* ≤ 0.05) for test samples compared to normal cells and nano-ZnO-treated cells without AHLs addition, respectively.

Nano-ZnO exposure for 6 h induced a significant increase (*p* ≤ 0.05) in SOD activity in *N. europaea* cells (Figure 4). The presence of C_6_-oxo-HSL at all tested concentrations from 0.01 to 1 µM significantly enhanced (*p* ≤ 0.05) SOD activity during 6 h of NP exposure, when compared with either normal cells or nano-ZnO-treated cells. Therefore, AHL addition remarkably enhanced cellular anti-oxidative stress capacities under nano-ZnO stress.

### 3.5. Nano-ZnO Dissolution

**Figure 5:**
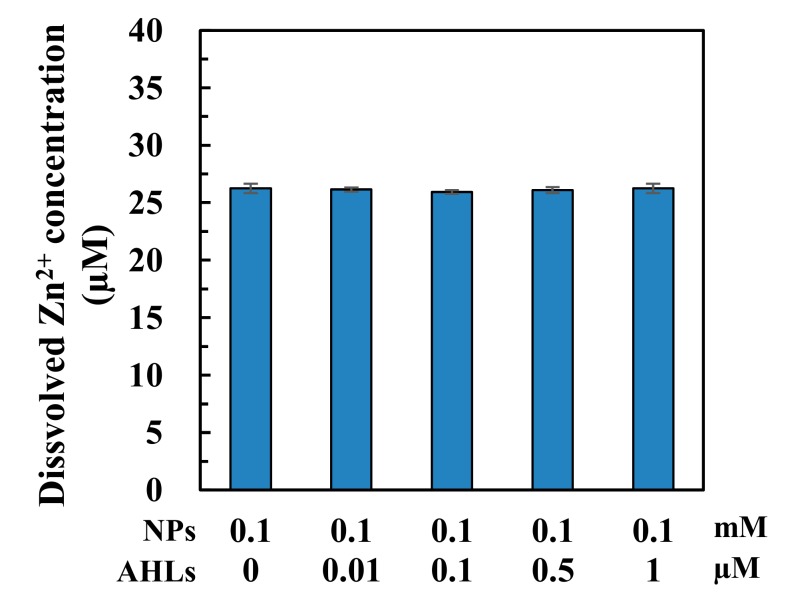
Solubility of 0.1 mM nano-ZnO in the *N. europaea* cultures with 0.01, 0.1, 0.5 or 1 µM *N*-acyl-homoserine lactones (AHLs) addition after 6-h incubation.

The dissolved Zn^2+^ concentration released from nano-ZnO are shown in Figure 5 when added with AHLs at 0.01–1 µM (containing DMSO accordingly at 0.003–0.33%). It can be seen that the dissolved Zn^2+^ concentration was around 0.026 mM in cultures when treated with 0.1 mM n-ZnO alone and was not significantly (*p* > 0.05, *t*-test) affected by AHL (containing DMSO) addition at any concentration tested (Figure 5). Therefore, exogenous addition of AHLs plus DMSO would not dramatically impact the nano-ZnO dissolution.

## 4. Discussion

The addition of the signalling molecule, C_6_-oxo-HSL, should help improve bacterial tolerance to nano-ZnO stress and an AHL dose of 0.01 µM significantly attenuated the NP-induced inhibition of cell proliferation, membrane integrity, specific AMO activity, and *amoA* expression in *N. europaea* (Figure 2 and Figure 3). Ag NPs have been shown to significantly decrease the C_6_-oxo-HSL concentration and interrupt QS regulation in the Gram-negative bacteria, *Pseudomonas syringae* and *Pantoea stewartii* [24]. The addition of AHLs might facilitate QS regulatory functions to enhance bacterial tolerance to nano-ZnO stress and thus rescue the inhibited metabolic activities of *N. europaea* (Figure 2 and Figure 3) by compensating for the potentially depressed AHL levels in the solution. This is supported by the findings that the addition of 0.01 µg/mL (~0.046 µM) C_6_-oxo-HSL into a starved *N. europaea* culture promotes cell growth through QS regulation during the recovery from starvation [20]. In addition, QS has been shown to mediate the bacterial adaptive responses to graphene oxide stress in biofilm microbial communities [23].

Nano-ZnO has been widely reported to exert bio-toxic effects through physical contact, dissolved Zn^2+^ release, or ROS production [5,9]. In the present study, dissolved Zn^2+^ (Figure 5) was expected to greatly contribute to the toxicity of nano-ZnO, despite the fact that the toxic effects of nano-ZnO on *N. europaea* cells were observed to be higher than those of Zn^2+^ in our previous study under the same test conditions [32]. However, Zn^2+^ release from nano-ZnO was not notably affected by AHL addition (Figure 5) and is, therefore, not likely to be responsible for the observed change in NP cytotoxicity. In addition, nano-ZnO was expected to induce ROS generation, even in the dark, although illumination generally increases ROS concentrations [33,34]. The generated ROS could induce oxidative stress and exert negative impacts on microbes, such as membrane impairment, DNA/RNA damage, lipid peroxidation, and enzyme activity inhibition [35]. The observed increase of SOD activity (Figure 4), which is actively involved in ROS quenching and antioxidant defences [36], after AHL addition, suggested the potential involvement of QS regulation in the oxidative stress tolerance enhancement in *N. europaea* cultures in response to nano-ZnO exposure.

*N. europaea* cells have been shown to fight against nano-ZnO toxicity through the transcriptional regulation of a diverse range of functional genes and the associated biological pathways [15,37]. The addition of 0.01 µM AHLs may have induced QS-mediated gene expressions to rescue the metabolic activities inhibited by nano-ZnO (Figure 2 and Figure 3). For example, diverse membrane metabolism regulation, including osmotic equilibrium adjustment, structure preservation, and membrane transport, are actively involved in the cellular tolerance and adaption to nano-ZnO exposure [37]. Exogenous supplementation of AHL might potentially induce the regulation of membrane metabolism to resist nano-ZnO stress and thus, alleviate the membrane impairment (Figure 2B). Besides, nano-ZnO-treated cells could activate the transcription of relevant enzymes, such as thioredoxin, DNA repair enzymes, and extracytoplasmic function sigma regulator, to alleviate ROS-induced oxidative stress [15]. In this study, AHL addition might also help improve cellular ROS-quenching capacity, as indicated by the enhanced SOD activity (Figure 4). Furthermore, the *amoA* gene, encoding AMO, was up-regulated by 7.3% after the addition of 0.01 µM AHLs (Figure 3C). Stimulation of *amoA* transcription is considered as an antioxidant response to overcome the loss of AMO activity under nano-ZnO and Zn^2+^ stress [15,38]. Therefore, AHL treatment probably improved the antioxidant potential of NP-stressed cells by regaining the depressed AMO and ammonia oxidation activities (Figure 3A,B).

AHLs displayed a dose-correlative impact on nano-ZnO toxicity, and increasing the concentration of AHL did not always favour the tolerance of cells to NP stress. Interestingly, exogenous dosing of AHLs at 0.1–0.5 µM only improved cell density and membrane integrity under nano-ZnO stress, while metabolic functions, including AOR, AMO activity and *amoA* expression, were still inhibited or even deteriorated further (Figure 2 and Figure 3). These results indicated that QS regulation for stress tolerance was dependent on the AHL dosing concentration. An increase in AHL concentration was expected to be immediately perceived by *N. europaea* cells, resulting in their coordination as a group [18] to fight against NP exposure. Thus, AHLs dosing at 0.1–0.5 µM were not expected to cause notable reductions in cell density and membrane integrity (Figure 2). However, Batchelor et al. [20] reported that increasing the concentration of C_6_-oxo-HSL decreases its growth- and activity-promoting effects on *N. europaea*. Therefore, it is possible that a higher dose of AHLs might compromise the QS regulation effects on *N. europaea* cells for NP stress resistance, such as the impaired AOR, AMO activity, and *amoA* expression seen in this study (Figure 3). With further increase of AHL concentration up to 1 µM, the alleviation of QS regulation effect might even exacerbate nano-ZnO toxicities, which finally resulted in the observed inhibitions on all cellular metabolic activities (Figure 2 and Figure 3).

The reason for the compromised QS regulation effects with increasing AHL concentrations observed by Batchelor et al. [20] and in this study remains unclear. One possible explanation is that *N. europaea* is generally an autotrophic bacterium that lacks key genes necessary for the transport and assimilation of organic substances [39], and it is sensitive to environmental perturbations, including the presence of organic compounds (Hallin et al., 2005; Radniecki et al., 2008; Urakawa et al., 2008). AHLs function not only as QS signals, but also as organic substrates. It has been reported that AHLs are assimilated or metabolized by heterotrophic bacteria in activated sludge [40,41]. As monocyclic organic compounds, AHLs at higher concentrations might potentially affect the metabolic and ammonia oxidation activities of autotrophic *N. europaea* cells, especially when they are stressed by nano-ZnO. The oxic ammonia oxidation rate of *N. europaea* cells has been shown to significantly decrease by approximately 45%, without bacterial growth, in the presence of the small-molecule organic compound, fructose [42]. Therefore, a higher dose of AHLs may compromise the effects of QS regulation on cellular tolerance to nano-ZnO or even exert toxic effects on cells.

## 5. Conclusions

This study preliminarily examined the effects of exogenous AHL on a typical ammonia oxidizer-*N. europaea* under nano-ZnO stress. AHL displayed a dose-correlative impact on cellular tolerance to nano-ZnO exposure, and AHL at a dose of 0.01 µM significantly alleviated the acute nanotoxicities. Moreover, AHL addition notably enhanced cellular anti-oxidative stress capacities. The findings in this study provide novel insights into the development of QS regulation strategies to alleviate NP toxicity and its environmental risk.

## Figures and Tables

**Table 1:** Effects of dimethylsulfoxide (DMSO) solvent (no *N*-acyl-homoserine lactones (AHLs) addition) on cell density, membrane integrity, and ammonia oxidation rate (AOR).

DMSO Content (*v*/*v*)	Corresponding AHLs Content (µM)	Cell Density (lg(mL^−1^))	Membrane Integrity (%)	AOR (10^−2^·mg N/L/min)
0% (Control)	0	8.49 ± 0.30	98.98 ± 0.07	9.07 ± 0.08
0.33%	1	8.45 ± 0.48 *	98.08 ± 0.23 *	8.58 ± 0.58 *
0.17%	0.5	8.46 ± 0.20 *	98.74 ± 0.43 *	9.04 ± 0.30 *
0.03%	0.1	8.48 ± 0.50 *	98.22 ± 1.35 *	8.63 ± 0.52 *

Note: ‘*’ means no significant difference (*p* > 0.05) in comparison to the control.

**Table 2:** Oligonucleotide primers for *amoA* and 16s rRNA genes and amplification programs used in quantitative reverse transcription polymerase chain reaction (qRT-PCR) quantification.

Target Gene	Primer Sequence	Length (bp)	Amplification Procedure
*amoA*	F: GGACTTCACGCTGTATCTG R: GTGCCTTCTACAACGATTGG	662	Pre-denaturation: 95 °C, 3 min Denaturation: 95 °C, 20 s Annealing: 59 °C, 30 s Elongation: 72 °C, 20 s Cycle: 40 Melting curve: from 55 to 95 °C, 0.1 °C/s Final hold: 4 °C
16S rRNA	F: TCCTACGGGAGGCAGCAGT R: GGACTACCAGGGTATCTAATCCTGTT	1462	

## Abbreviations

MNPs	metallic nanoparticles
nano-ZnO	ZnO nanoparticle
BNR	biological nitrogen removal
AOB	ammonia-oxidizing bacteria
QS	quorum sensing
AI	autoinducer
3-oxo-C_6_-HSL	*N*-3-oxo-hexanoyl-homoserine lactone
AHL	*N*-acyl-homoserine lactone
AOR	ammonia oxidation rate
DMSO	dimethylsulfoxide
DO	dissolved oxygen
SOD	superoxide dismutase
AMO	ammonia monooxygenase
qRT-PCR	quantitative reverse transcription polymerase chain reaction
